# Sustaining the urban commons in Ghana through decentralized planning

**DOI:** 10.1016/j.heliyon.2023.e15895

**Published:** 2023-05-04

**Authors:** Mohammed Abubakari, Abdul-Salam Ibrahim, Benjamin Dosu, Mudasiru Mahama

**Affiliations:** a Huts and Cities Ghana Limited, Accra, Ghana; b School of Environment and Sustainability, University of Saskatchewan, Saskatoon, Canada; c Department of Geography and Planning, University of Toronto, Toronto, Canada; d Global Youth Innovation Center LBG, Sunyani, Ghana; e Sahtú Renewable Resources Board, Tulita, NT, Canada; f Department of Geography and Sustainability Science, University of Energy and Natural Resources, Sunyani, Ghana; g SD Dombo University of Business and Integrated Development Studies, Wa, Ghana

**Keywords:** Urban planning, Urban commons, Sustainability, Institutional analysis and development (IAD) framework, Non-cooperative game theory, Ghana

## Abstract

With global urbanization on the increase and cities now hosting more than half of the planet's population, there are concerns regarding the protection of urban commons as part of sustainability efforts, especially in sub-Saharan Africa. Decentralized urban planning is a policy tool and practice that is used to organize urban infrastructure for sustainable development. Yet, how it can be used to sustain the urban commons remains fragmented in the literature. This study reviews and synthesizes urban planning and urban commons literature using the Institutional Analysis and Development Framework and the non-cooperative game theory to identify how urban planning can protect and sustain the urban commons – green commons, land commons, and water commons – in Ghana. The study, based on the determination of different theoretical scenarios for the urban commons, identified that decentralized urban planning can help sustain the urban commons, but it is operationalized in an unfavorable political environment. For green commons, there are competing interests and poor coordination amongst planning institutions, and the absence of self-organizing bodies in managing the use of such resources. For land commons, increased land litigations are characterized by corruption and poor management of land cases in the formal land courts, and despite the existence of self-organizing institutions, these institutions have failed to act responsibly to protect land commons due to the increasing demands and values (profitability) of lands in urban areas. For water commons, urban planning has not been fully decentralized and there is also the absence of self-organizing bodies in urban water use and management. This is coupled with the waning of customary water protection provisions in urban centers. Based on the findings, the study generally proposes institutional strengthening as the bedrock for enhancing the sustainability of the urban commons through urban planning and should therefore be of policy focus moving forward.

## Highlights

• We examine how decentralized urban planning can be used to protect and sustain the urban commons in Ghana.
• We develop theoretical scenarios using the IAD framework and the non-cooperative game theory.
• Commons sustainability is limited by an unfavorable political environment.
• Strengthening decentralized planning institutions will improve commons sustainability.

## 1 Introduction

Urban commons^1^ have become more vulnerable due to incessant urbanization, climate change, migration, and the transition toward more competitive environments on a global scale [[1], [2], [3], [4]]. With cities dealing with the repercussions of economic disparity, ethnic multiplicity, and uneven spread of environmental risks, there has been a steady erosion of urban commons through privatization [5]. In sub-Saharan Africa (SSA), the urban commons are held in high esteem by most traditional communities. In recent times, however, urban commons have come under varied contestations in the sub-region, mirroring the shared cultural sensitivity of the people and their dependence on them as a source of livelihood [1,6]. Consequently, the dilemma of the urban commons in SSA is multifarious, involving a multiplicity of actors [6]. Moreover, the next face of urbanization is globally projected to be more pronounced in the developing world, including SSA, hence accentuating the necessity of exploring different viable alternatives for safeguarding the urban commons [7].

In protecting the urban commons in the SSA region, the significant role of decentralized urban planning has generally been acknowledged in policy and academic discourses [8]. The United Nations Sustainable Development Goal (SDG) - 11, for instance, emphasizes the need to make cities and human settlements inclusive, safe, resilient, and sustainable [7], especially in developing regions such as the SSA. Eidelman and Safransky [9] contend that the urban commons can be used to conceive strategies to build inclusive and sustainable cities through decentralized urban planning. However, Alipour and Arefipour [10] have observed that planning the (urban) commons is typically devoid of sustainability corridors for posterity. Specifically, as some authors [8,11,12] observed, unlike in the developed world where a conscious consideration of the (urban) commons in decentralized planning is apparent, its manifestation in regions such as SSA is rare.

The literature on the urban commons in the SSA region, on the one hand, has largely focused on decentralized common property management and sustainability within the context of rapid urban expansion [[13], [14], [15], [16], [17]]. For instance, Foster [17] explored the sustainability of the urban commons through collective action, and Esopi [18] examined how the resilience of the urban commons can increase the value of the urban system. The study of Esopi [18] identified that rapid changes in cities, triggered largely by economization, make the urban commons somewhat fragile. Resultantly, this creates a conundrum for urban institutions responsible for sustainable development, especially those in SSA that are predominantly under-resourced. On the other hand, urban planning studies have largely focused on urban transport planning, land use planning, energy planning, spatial planning, and sanitation planning and their contribution to sustainable development [[19], [20], [21], [22]]. Notwithstanding, such urban planning studies unintentionally provide some useful insight into using decentralized planning to sustain the urban commons, adding to other studies geared toward urban planning and the urban commons. The growth in urban planning and urban common literature has been largely fragmented to properly inform policy science for protecting the urban commons using decentralized planning as a policy tool, instrument, and practice. This calls for a study that synthesizes the fragmented literature on urban planning and urban commons to guide and direct future studies in the SSA region, specifically, Ghana, hence the focus of this paper.

In Ghana, the manifest threat of urbanization and climate change to the sustainability of the urban commons has sparked debate in policy and academic cycles about the routes to settling the dilemma of the urban commons. Debrah et al. [23] examined green commons within the framework of a smart and sustainable built environment and argued for planning and policy interventions to be centered on the pillars of sustainability for the management of green resources. Ibrahim et al. [13] looked at land commons and suggested that the self-organized institutional arrangements should be incorporated into the decentralized land governance system in Ghana. Twum and Abubakari [24] studied water commons and private sector involvement and called for strict State regulations and community-initiated water supply systems in planning for urban water needs in Ghana. These studies give an indication that decentralized urban planning could then be used as one of the tools to safeguard urban commons. This study (based on the traditional literature review approach) theoretically reflects on the Institutional Analysis and Development (IAD) framework and the non-cooperative game theory [see 25] to propose institutional restructuring and reforms for decentralized planning of the urban commons in Ghana. The aim is to identify how urban planning can be transformed and subsequently positioned to protect and sustain urban commons focusing on green commons, land commons, and water commons in Ghana. The findings of the study are expected to have implications for urban planning practices in contributing to the sustainability of the urban commons in SSA.

In the subsequent sections of the study, we have presented the approach we used for the literature review as well as a framework that depicts how decentralized urban planning can help sustain the urban commons. This is followed by theoretical literature on commons sustainability focusing on the IAD framework and the non-cooperative game theory for the conceptualization of the findings of the study. Following this, we have discussed the main findings before concluding the study with some useful measures to streamline urban planning for sustainable urban commons in Ghana.

## 2 Literature review approach

This study is a desk review aimed at connecting two streams of scholarly works, urban planning literature on the one hand, and urban commons literature on the other hand to provide ways in which decentralized urban planning can be used as a conceptual and policy tool and practice to protect the urban commons in Ghana. As a result, we synthesized different as well as complementary literature which can also serve as a foundation for future empirical works that will explore the nexus between urban planning and urban commons in Ghana and SSA broadly.

We obtained secondary documents from Scopus, Web of Science, Google Scholar, and Google. For Scopus and Web of Science, the following search queries were used to search for documents: “Urban Commons” AND “decentralized urban planning” OR “urban planning” OR “Ghana”; “Green resources” AND “urban planning” OR “urban Ghana” OR “decentralized planning”; “Land” AND “urban planning” OR “urban Ghana” OR “decentralized planning”; and “Water resources” AND “urban planning” OR “urban Ghana” OR “decentralized planning”. A number of documents (about 20 publications) were obtained through the search in Scopus and Web of Science, and these documents were complemented with a search in Google Scholar and Google using the following descriptions: “Decentralized urban planning and urban commons in Ghana”; “Sustaining urban commons in Ghana through decentralization and planning in Ghana”; “Decentralized urban planning and urban commons sustainability in sub-Saharan Africa”; “Managing green resources as commons in urban Ghana”; “Managing land as a commons in urban Ghana; and “Water resources as commons and their sustainability in urban Ghana”. Over 40 publications were obtained in both Google Scholar and Google, in which some were excluded (those whose aims did not corroborate with the objective of the study). For the documents that were included, their references provided additional literature that was relevant to the study.

For the theoretical literature of the paper, we were aware of the defining scholarly works of Vincent and Elinor Ostrom and their colleagues in commons scholarship. As a result, we specifically searched for their works and related ones such as that of Garrett Hardin and other commons scholars. One key material we considered was Elinor Ostrom and colleagues’ book entitled “*Rules, Games, and Common-Pool Resources”* [25] where we learned more about the (Urban) commons, the IAD Framework, and game theories. In all, over 100 publications were gathered, reflected upon/analyzed, and organized/synthesized to inform the writing and referencing process of the study.

## 3 Decentralized urban planning and the sustainability of the urban commons in Ghana

Urban planning encompasses a broad spectrum of interventions, including urban design and reconstruction, as well as urban development with attention granted to politics of development, power manifestations, institutions, and their levels, as well as the relationships that shape urban forms in a desirable manner [[26], [27], [28]]. Scientific commentaries on urban planning have largely positioned urban individuals, including traditional leaders, politicians, citizens, and real estate developers at the center of the change process in urban spatial development [29,30]. Thus, the success of decentralized urban planning in sustaining urban commons connects greatly with how institutions and citizens are engaged in the process. In this regard, Cobbinah and Darkwah [31] are of the view that adequate institutional-resource provision for decentralized urban planning bodies is a prerequisite in the SSA, specifically Ghana. In the same context, Njoh [32] and Pløger [33] have commonly advocated for a rightful participatory regime, where all concerned actors and institutions are involved in taking rightful planning decisions. In Ghana and SSA, limited resources for decentralized urban planning bodies are one major setback, and this manifests in the context of the opaque participation of urban members in the urban planning process. Gunder and Mouat [34] blame the ineffective participation of urban members as partly driven by the dominance of politicians’ abusive use of power. This situation, characterized by rapid urbanization [35,36], makes the sustainability of urban commons through the use of planning a complex puzzle in the SSA. As a result, urban commons, in particular, green commons, land commons, and water commons are either misused, over-used, contested, or misplanned.

Focusing on green commons, Debrah et al. [23] identified barriers such as poor policy implementation, the lack of awareness of the benefits of green resources, increased generation of waste, and improper management, as well as environmental degradation. Hence, green commons are continuously sacrificed instead of being managed to meet the needs of the growing urban population. Amoako and Adom-Asamoah [37], in observing the situation, remarked that the sustainability of green resources in the urban setting is a difficult task for planning authorities. This may thwart the attainment of SDG-11 “making cities and human settlements inclusive, safe, resilient, and sustainable” [see [38], p. 1–6]. Furthermore, whilst urban greening and forestry, which concerns itself with the establishment, promotion, maintenance, and management of forest resources, including trees in urban centers and their fringes [39], has gained prominent attention in developed countries, the case in SSA is entirely different as urban greening and forestry practices are fragilely practiced. This situation threatens policy and planning interventions for the sustainability of urban green commons.

Land as urban commons also remains an instrumental resource for (urban) development [40], as they serve as a livelihood source [41] and an avenue for urban spatial development. Notwithstanding, sustainable land management for the collective good of the urban populace, through the aid of decentralized urban planning, has been challenged due to conflicts amongst urban members [42,43], with outcomes leading to poor land-use planning [31].

Urban water commons such as community wells, rivers, dams, and rainwater are hardly considered relevant in urban planning processes in SSA. Rapid urbanization in SSA has driven informality, high densities, and congestion along urban water commons such as rivers, which greatly contributes to disasters such as flooding [44]. Decentralized urban planning agencies have not been able to control and regulate the use of urban water commons as well as the physical development around them. As a consequence, some resource users mismanage water resources to the disadvantage of others. Whilst urban planning has generally failed in the management and sustainability of water resources, it is still seen as a great tool that can be used to complement the effective governance of (urban) commons for the sustainable development of Ghana and SSA [45].

## 4 Establishing the linkages: commons' theoretical thoughts, institutions, and the IAD framework for urban commons' sustainability

### 4.1 Relevant theoretical reflections on the commons

Urban commons are resources that are natural, typically communal, and characterized by a non-exclusive property right in urban centers. These attributes of the (urban) commons validate the need to safeguard them for posterity [46]. Commons are often distinguished from other resources/properties such as private properties, public properties, and toll properties [25], but in most cases, regarded as open-access resources. Commons as open-access resources are subjected to tragedy [47], as they could suffer from over-exploitation from self-seeking actors. Hardin [47] contended that the private property rights regime, when transcended to the commons, can possibly help in reversing the commons' tragedy. This corroborates with the general acknowledgment within the commons scholarship that in most instances, common property rights are converted to private property rights due to increasing population pressure on the commons [48]. On the other hand, Ostrom [49] observed that groups sharing access to the commons could instead self-organize to govern the resource system in a manner that prevents the tragedy that usually befalls such resources. However, to achieve effective self-organization, Foster and Iaione [1], and Frischmann et al. [50] recommend the existence of institutional arrangements to regulate the benefit-sharing emanating from the resource system for the collective good. In an analysis of Hardin's perspective in relation to Ostrom's, Soedomo [51] concluded that their respective perspectives could be supported by the same theory. Also, both Soedomo [51] and Ostrom [52] have recommended effective monitoring of commons as well as the creation of platforms for resource users to regularly interact to increase mutual trust.

Historically, commons theorists have largely based their conclusions on evidence from the countryside [9], hence making the analysis of the urban commons imperative. There are two schools of thought in the analysis of the (urban) commons. The first school views the commons as non-subtractable resources in the urban space whose consumption does not deplete the amount available for other users [see, for instance, [18, 53]]. Here, the urban commons are contextually characterized by idiosyncrasies, including contiguity, compactness, and multifariousness that make their consumption fruitful for improving the value of the urban system [54]. Thus, urban commons are continuously being produced through the interaction of diverse forces within the urban setting [55]. This definition expands the urban commons to include (inter alia) civic spaces, including playgrounds, streets, recreation areas, parking spaces, community parks, gardens, urban public spaces, streets, public roads, recreation areas, football fields, and basketball courts [56]. With this school of thought, the urban commons will only suffer from the tragedy of the commons at the point described by Foster [[17], p.66] as “regulatory slippage”. In this direction, local authorities, out of dereliction, can create room for resource users to exploit the commons by reducing its value to the detriment of other users [17]. The second school [see, for instance, [25, 49]] views the (urban) commons as subtractive resources whose consumptions reduce the amount available to others, making the use of such resources non-excludable and rivalrous. Here, the (urban) commons can potentially experience tragedy through exploitation and depletion [47]. Based on both schools of thought, the (urban) commons will inevitably experience over-exploitation unless regulatory measures are enforced. An enhanced appreciation of the (urban) commons will allow for their integration into the decentralized (urban) planning context [57], having regard to the condition of the resource, the costs of management, and the derived benefits of the resource.

### 4.2 Institutions and rules, and the game theory for understanding commons' dilemma

In commons research, institutions are “enduring regularities of human action in situations structured by rules, norms, and shared strategies, as well as by the physical world” [[58], p.582]. They entail a set of prescriptions that are created by humans for the organization of all forms of interactions, either repetitive or structured, including those involving governments at all levels [59]. People behave in a certain manner in their interactions due to institutions; as Hodgson [60] captured it, institutions become the “stuff of life”. Rules are “generally agreed upon and enforced prescriptions that require, forbid, or permit specific actions for more than a single individual” [[61], p.250]. The theory that can resonate from the complex interactions and the rules that govern them are central to resolving complexities and dilemmas in commons research. The non-cooperative game theory, through the lens of rationality, can provide an interface to understanding actors and their actions within a ‘common’ environment, and in furtherance, aid predictions into the future [25]. In this sense, the institutions and rules can be viewed to manifest in the form of a game, comprising of different elements – (i) players; (ii) positions; (iii) actions for each position and chance moves; (iv) decision mapping into outcomes; (v) the outcomes themselves; (vi) the information available; and (vii) subsequent payoffs depending on costs and benefits of actions and outcomes – to help analyze action situations (these are the compositions of the IAD framework, which have been espoused in the immediate subsection). We consider the urban settings of Ghana as a field composed of non-cooperative actors, and their interactions can be understood through the lens of the formal non-cooperative game theory and with the aid of the IAD framework [25].

### 4.3 Urban commons’ sustainability through the lens of the IAD framework

The IAD framework is a solution-oriented multi-tier framework that outlines how institutions emerge, operate, and change over time for commons management [62]. Since commons are inherently communal, Heikkila and Anderson [63] note that interested actors could self-organize a design and adapt institutions through local institutional arrangements to manage the commons to guarantee their sustainability. This local institutional structure can be attained either through independent community collective action or in collaboration with the local State institutions [10]. The IAD framework has three levels of analysis or arenas of choice, espoused by McGinnis [64]:

(i) “Constitutional Choice” - where the rules of engagements are defined, including the legitimate constitution of relevant collective entities involved in collective or operational choice processes.
(ii) “Collective Choice” - where the institutional structure is established based on the procedures set out by constitutional choice processes. This is also the level at which Ostrom's design principles (well-defined boundaries of resource and users; resource 'users' participation in rulemaking; monitoring and monitors' accountability to resource users; graduated sanctions; low-cost, accessible conflict resolution mechanisms; and nested institutional arrangements) are applicable.
(iii) “Operational Choice” - where specific actions are taken by legitimately authorized actors to address collective problems.

Our analysis and theoretical discussions focus on the second level, “Collective Choice,” whilst our proposals align with the third level, “Operational Choice”, involving the specific actions that urban planning agencies should pursue in using decentralized planning to protect the urban commons. The different arenas of choice highlight the intricacies involved in structuring a legitimate institutional structure for the sustainability of the (urban) commons. However, the framework acknowledges the intricacies that structure the institutional choice process by elucidating actors' idiosyncrasies that define their choices and conceptualizes the institutions that emanate from and shape the process, and the contextual variables that condition the interactions and outcomes of actors [63].

Central to the framework are “action situation” and “actors”. These two comprise an action arena. The action situation is a space for stakeholder engagements and the resolutions of the problems of the commons. Action situations are “the social spaces where individuals interact, exchange goods and services, solve problems, dominate one another, or fight” [[52], p.11]. The action situation is thus, made of participants (for instance, actors who play a role in a specific urban commons regime); positions (place-holders that associate participants with actions and outcomes); actions (for instance, the decision to exercise physically in an urban green open space, or fish in an urban water source); potential outcomes (what is affected as a result of participants’ activities such as the extent of damage to an urban water source or a mismanaged urban land); transformation functions (these map participants in terms of their decision to a set of outcome; for instance, urban dwellers in the same position can rally against actions of urban planning decisions); information available to a participant in a position at a particular stage (this can induce the actions of the participant in a particular way); and payoffs (could be benefit or cost of actions/outcomes such as eviction of urban dwellers). These variables permeate the “discussion section” of the study where we have developed different theoretical scenarios for the urban commons.

Within the context of action situations, actors are participants who have preferences, information processing capabilities, selection criteria, and resources [25]. These characteristics make actors extremely important, just like the action situations, thus, warranting special attention granted to actors to have a better understanding of commons dilemmas. The participation of actors in the action situation is largely driven by the expected benefits or costs associated with the different strategies and the possible outcomes [65]. The “rules-in-use”, and “biophysical and community context” shape the action situation by structuring the strategies that actors adopt. Biophysical and community contexts define the changing characteristics of the resource system and the communal responses (strategies) to the changes in the action situation. Actors may change their strategies upon understanding the outcomes of their earlier actions. This may determine the sustainability of the actions taken and the resource system. Ostrom [52] emphasized the creation of successive platforms for continuous actors' engagements to improve the outcomes of successful long-term governance of the resource system.

The IAD framework emphasizes the need for collaborative decision-making especially involving actors with inalienable attachments to the resource system and instituting an effective monitoring regime with a well-defined institutional network [63]. Also, the framework outlines “evaluative criteria” for the outcomes and the process of attaining the outcomes. These criteria include efficiency, fiscal equivalence, re-distributional equity, accountability, conformance to the values of local actors, and sustainability [52]. Whilst Ostrom [52] referred to the “evaluative criteria” as the analyst's verdict of the outcomes, McGinnis [64] described it as the actor's assessment of the outcomes of the action situation. We are of the view that analysts seeking to evaluate the sustainability of the (urban) commons should not only focus on the “sustainability criteria” since the manifestation of the other criteria, for instance, “efficiency” and “accountability”, can influence the sustainability of the process and the outcomes thereof.

**Fig. 1:**
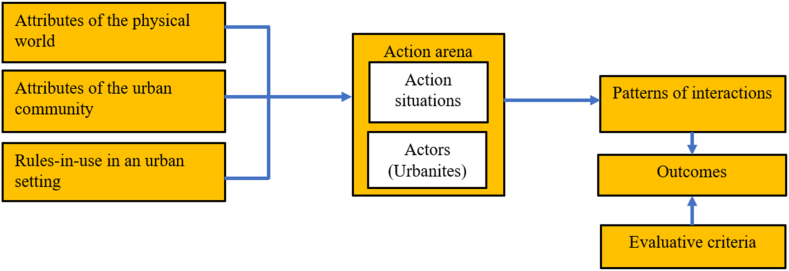
A framework for institutional analysis of the urban commons (Figure source [[59], p.15]).

The framework (Fig. 1) posits that communities must drive the initiation and maintenance of the institutional structure for commons governance. Within the urban context, however, communities cannot exclusively execute this agenda as the State is essentially ubiquitous in the city space and responsible for urban planning and design. The responsibility of urban planning is entrusted to the hands of (decentralized) State institutions. It becomes necessary for local actors to collaborate with State institutions for a robust urban planning intervention to safeguard the commons. The urban setting is “already congested, heavily regulated, and socially and economically complex” [[1], p.4]; so, the IAD framework cannot be wholly adopted in the urban context as some of its key elements could be lost in translation. This underpins the use of non-cooperative game theory to complement the IAD framework.

## 5 Decentralized urban planning and the urban commons in Ghana

### 5.1 Decentralized urban planning: challenges and prospects

**Fig. 2:**
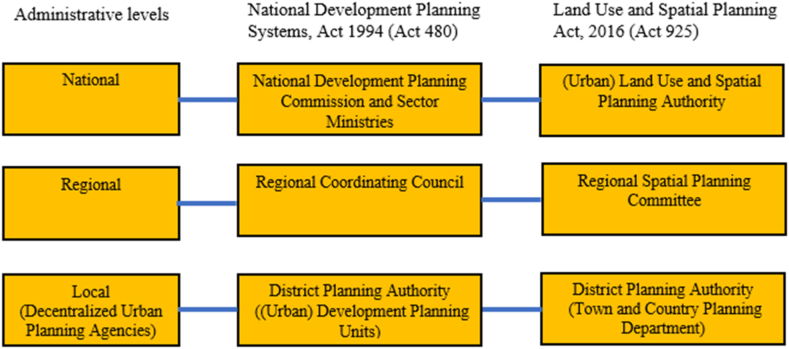
(Urban) Planning institutional arrangement in Ghana (Figure source [[79], p.46]).

Urban planning is an important tool to address urban development challenges in Ghana and globally. Sustainable urban development can be achieved when interventions reflect urban planning reasoning and align with the needs and aspirations of urban members [26]. The contemporary decentralized urban planning in Ghana emerged in 1988 when decentralization was adopted as the pathway to pursue development. This was spearheaded through the notion of bringing the government down to the local people and ensuring that government takes into consideration the perspectives of local members in pursuing planning and development agenda. The Local Government Act [66] was thus, introduced, and this was further augmented by other urban planning legislations, including the Land Use and Spatial Planning Act [67]; the Spatial Development Framework [68]; the National Urban Policy Framework [69]; and the National Development Planning Systems Act [70]. The Land Use and Spatial Planning Act [67] and the National Development Planning Systems Act [70], respectively, establish institutional arrangements for spatial (urban) planning, and (urban) development planning in Ghana (Fig. 2). Whilst (decentralized) urban planning is endorsed as one prominent tool the government can use to manage urban development [71] for inclusive growth in Ghana, it is, unfortunately, a reflection of political hegemony characterized by frequent planning errors that put ordinary urban members into persistent threats [[72], p.26]. As Cobbinah and Darkwah [31] captured, decentralized urban planning problems are usually passed to and managed by politicians (driven by power interest) [see also [73]] and traditional authorities with no technical understanding of how urban planning should function. As an outcome, the decentralized urban planning agencies exist as ‘white elephants’ that are steered around by the political system at different levels in a manner that meets the interest of the political elites and private actors to the disadvantage of the urban masses [74]. The dominance of traditional authorities over decentralized urban planning agencies, for instance, is caused by the commanding customary land ownership structure in urban Ghana, as customary authorities own about 80% of lands in Ghana [75,76]. Consequently, land allocations are mostly done by the traditional authorities with little or no consideration of planning procedures and methods [77]. This has rendered the ineffectiveness of decentralized urban planning as a prominent tool for urban development [78].

As a tool, Campbell and Fainstein [28] suggest that (decentralized) urban planning in Ghana can realize its role when it pays credence to asynchronous and symbiotic relationships in the development of urban regions. Resources and governmental support, as well as recognition of the central role of decentralized urban planning, are requisite [73] if the prospects of decentralized urban planning are to be ascertained. These prospects of urban planning lie in its focus [31]. The focus should be less on the scientific discovery of the best technical solutions, and more on processes of bargaining, negotiation, and compromise over how scarce environmental resources are distributed in a manner that promotes ‘common’ interests. Adding to that, Watson [45] calls for a pro-poor approach, riding on inclusivity and equitability as the centerpieces of urban planning. Currently, Ghana has divided its administrative regions into sixteen to further strengthen decentralized urban planning functions for the benefit of the local urban members. There are establishments of decentralized urban planning agencies across all the regions and districts to ensure a bottom-up urban planning approach.

### 5.2 Decentralized urban planning and the urban commons in Ghana

#### 5.2.1 Urban green commons

Urban green commons are critical environmental resources that promote ecological stewardship, socio-economic growth, and the general well-being of urban communities [80,81]. These benefits underscore the value of green commons/resources in the urban context [82]. While planning is instrumental in such regard, urban members as resource users must also be concerned with managing green commons in their neighborhoods and communities. This is essentially needed, as urban green commons in Ghana are continuously waning because of encroachment, rapid population growth, and infrastructural development that lead to the depletion of such resources [83]. Such resources are consistently in tragedy due to the dilemmas in their effective governance; urban planning itself is ineffective as institutions spearheading that are weak [31,37,84], whilst the urban members tend to display an adamant attitude in terms of their responsibility of taking good care of such resources [85]. The depletion of urban forest resources, for example, could also be related to the westernization of the cultural systems that respected forests as sacred, governed, and essentially sanctioned by spiritual deities [86]. Dumenu [87] revealed that the Achimota forest in Accra, the national capital, and one of the prominent green commons in Ghana, has severely depleted from its initial 500 ha–360 ha as a result of encroachment by some urban members. Also, Quartey [88] observed that the green commons that characterized Kumasi, the second-largest city in Ghana, have also been depleted, leaving only marginal forest areas in the city. The persistent depletion of urban green commons comes with a severe economic cost that affects the required resources for their maintenance [89].

In the decentralized urban planning context, the Metropolitan, Municipal, and District Assemblies (MMDAs) with support from the Land Use and Spatial Planning Authority (LUSPA); the Land Commissions; the Department of Parks and Gardens (DPG); the Environmental Protection Agency (EPA); and the Forest Commission (FC) are expected to streamline planning interventions to protect urban green commons [90]. However, competing interests from other institutions such as traditional authorities and political agencies, and poor coordination (can) impede effective decentralized urban planning interventions [37]. The absence of self-organizing bodies amongst urban members has also rendered the efforts of external institutions such as environmental civil society organizations less impactful for sustaining urban green commons in Ghana.

#### 5.2.2 Urban land commons

Land is an asset and the foundation of Ghana's resource base and serves as the main source of livelihood for people [91]. The intense demand for land commons has increased their values, making these resources to be leased for profit by chiefs or clan heads who are the allodial title holders [77]. As a result, the livelihoods of some Ghanaians who depend on land commons are affected [92]. Sometimes, members of land ownership families who are expected to manage their ‘common’ lands for the collective good disagree with the decisions of their chiefs or family heads, leading to a multiplicity of conflicts around such lands [93]. According to Kludze [94], land-related litigation has completely overwhelmed the Land Sector Agencies (LSAs), and formal land courts are hampered by poor case management, corruption, and shortages. All these problems relating to land commons, especially in urban settings, have severely affected urban land use planning interventions for development.

Following the problems surrounding land commons, arguments have mounted in the direction of land administration, where some contend that land commons should be administered by the State [41,95] whilst self-organizing institutions led by traditional leaders should simply have ownership rights, without any power to administer their ‘own common’ lands. Though (urban) land use planning has been weak in Ghana, it could be instrumental in land-use identifications that support sustainable development in urban communities [96]. Decentralized Urban planning institutions responsible for land use planning in Ghana are the Metropolitan, Municipal, and District Assemblies (MMDAs)/District Planning Authorities (DPAs); the Physical Planning Department; the Lands Commission; and the Traditional institutions.

#### 5.2.3 Urban water commons

Ghana is endowed with water resources through three main river systems. They are the coastal basins river systems, the southwestern basin, and the Volta basin respectively covering 8%; 22%; and 70% of the total area of Ghana. There are also groundwater resources in both the sedimentary rock formation of Voltaian origin (occupying 43% of the total area of Ghana, with estimated yields of 1.0–12.0 cubic meters per hectare at a depth of 20–80 m), and the non-sedimentary formation composed of crystalline basement complex (occupying 57% of the total area of the country with yields of 1.5–32.0 cubic meter per hectare at a depth of 20–100 m) [97]. The water resources are currently experiencing depletion. It is estimated that water stress will hit Ghana by 2025 [98]. Historically, the management of (urban) water resources has been spearheaded through customary provisions. Opoku-Agyeman reiterated that the ancestors were believed to ‘watch over’ water resources to ensure their sustainable use [99]. In some communities, including the urban centers, local members once believed in the existence of deities in water bodies that define rules and customs for the protection of water resources [86,100]. However, the emergence of Westernized religion and traditions has thwarted the beliefs and acceptance of customary provisions [101]. This has induced unwarranted behaviors and practices such as pollution, especially in urban centers.

Uncontrolled urbanization is also adding to the management dilemma of water commons [102], posing a severe threat to access to potable water for basic household needs. It is, therefore, vital that efforts are made to ensure an adequate supply of water in urban Ghana [103] through effective planning interventions toward water commons. Unfortunately, decentralized urban planning has largely neglected water, as it focuses more on land and infrastructural management. Water supply, for instance, has not been fully decentralized to ensure the integral role of local planning authorities in the management of water resources and their supply in their areas of jurisdiction [104].

Some urban members depend on water resources for some of their basic household activities such as washing and for socio-economic activities such as backyard farming, gardens, and other forms of urban agriculture. Notwithstanding, the urbanites have largely polluted water resources by channeling their drainage and waste into them. For instance, Asumadu-Sarkodie et al. [105] identified that the Korle Lagoon located in Accra has become a source for the disposal of large quantities of untreated industrial and household wastes, causing severe pollution and distortion of the natural ecology of the lagoon. Owusu Boadi and Kuitunen [106] also added that the pollution of the lagoon has significantly contributed to urban flooding, as the lagoon easily overflows when it rains. Water resource planning interventions are highly centralized in Ghana. The Ghana Water Company (GWC), the Water Resource Commission (WRC), the Environmental Protection Agency (EPA), and Irrigation Development serve as the key actors for sustainable urban water management in Ghana.

## 6 Theoretical discussions

As identified in the literature, there are discrepancies in urban planning as a tool to contribute to sustaining the urban commons in Ghana. In this section, we discuss urban commons governance and management complexities in Ghana through the IAD framework and the non-cooperative game theory [see [25]]. Before proceeding to the fundamental argument, we agree with Watson [45] that effective urban planning can play a critical role in the protection, management, and even expansion of urban commons, especially green commons. Thus, our discussion lies on the premise that institutional strengthening can form the backbone for enhancing the sustainability of urban commons through decentralized urban planning in Ghana. Proposals gathered from the literature in that direction are thus synthesized in the conclusion section of the research. The discussion largely focuses on “action situations” and “actors” surrounding the urban commons (green commons, land commons, and water commons) to theoretically reflect the tragedies faced by these commons in Ghana.

Hardin [47] has indicated that self-seeking resource users can take advantage of their user rights to over-exploit and mismanage commons to meet their selfish interests without recognizing the consequences of their actions on other members. Ostrom [49] has also alluded to the relevant role of self-organizing institutions to ensure the effective management of the commons, but in the Ghanaian context, these organizations are extremely weak, and in some instances, they hardly exist, especially with regard to green commons and water commons. External supportive institutions such as local authorities are also weak, leading to “regulatory slippage” [17] of the urban commons. The IAD framework differentiates three levels that define choices—"Constitutional, Collective, and Operational choices”. Whereas the “constitutional choice” level has defined formal rules to guide collective and operational choices, these rules are hardly followed due to weak urban planning agencies that must enforce them. This situation, in the context of very weak self-organizing institutions, has, at the “collective choice” level, made rules to be hardly followed, resource monitoring ill-effective, and sanctions almost never applied to urban commons’ distorters.

At the “operational choice” level where specific actions should be taken by responsible, legitimate institutions, the requisite actions such as evictions and conviction of victims are hardly pursued for the collective good and for commons sustainability due to the political interest of the ruling elites to safeguard their positions. When misplaced political interest is placed ahead of policies and regulations of the commons, sustainability is threatened. Regular interactions amongst resource users are important in the effective management of commons [51,52], but they hardly exist in the urban settings of Ghana, which are large geographical areas with a large number of people who hardly get time to meet and discuss issues that affect them [107]. Urban poverty is also prevalent [108], and so, everyone is seeking ways and means to make ends meet, whether or not their actions and inactions have negative outcomes on the sustainability of the urban commons. However, strong and committed decentralized institutions and political commitment can bring actors together to foster the development and maintenance of the urban commons for sustainability [109].

Institutions and rules could possibly be viewed in the form of a game [25]. Thus, we use the non-cooperative game theory to ‘model’ our analysis. We are aware of the quantitative complexities of the theory, but we use the basic ideas of the theory. In doing so, we assess the possible behaviors of resource actors so that useful remedies and implications are made to achieve the objective of the paper. We cut down the complexities in the use of the game theory by treating ‘homogeneous individuals’^2^ (for instance, urban farmers) as one “player” in the game instead of individual members. This approach has thus made the theoretical discussion less complex and easy to follow and understand. In this consideration, we see all urban members who are users of the urban commons as rational beings - decisions made by them are based on rational thinking. The elements (participants, and strategy) of the action situation are, therefore, relevant to be examined in the context of a game. Considering the first element, “the participants”, otherwise considered as “players” in the game, we see different groups of urban members are “players” who make single decisions simultaneously and independently and can take one or more actions, herein considered “strategy”. We have developed three different theoretical scenarios which have been determined based on how the specific urban commons considered in this study are generally managed in Ghana.

**Fig. 3:**
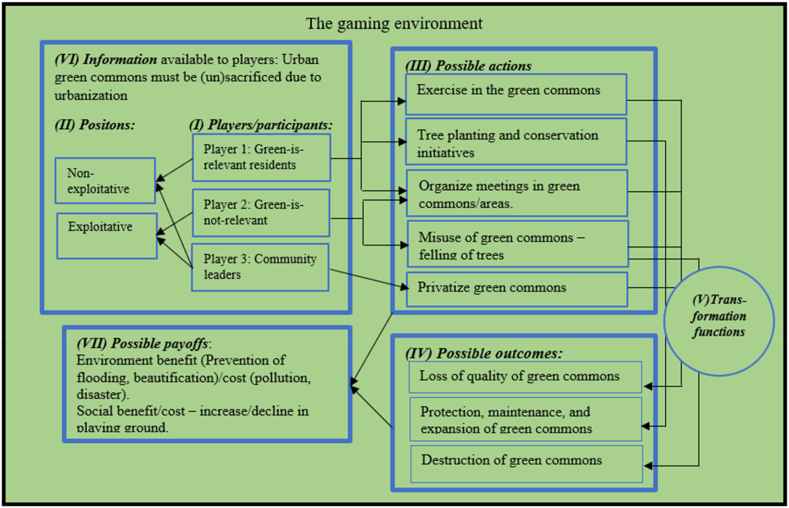
The game of urban green commons through the lens of the IAD framework. (For interpretation of the references to colour in this figure legend, the reader is referred to the Web version of this article.)

In the context of urban green commons, the following set of “players” are considered--"Player 1: urban residents” who see green commons as useful - Green-is-relevant residents (joggers, gardeners); “Player 2: urban members who do not really recognize the usefulness of green commons” - Green-is-not-relevant residents (poachers); and “Player 3: community leaders”. The positions of these “players” could be “exploitative” or “non-exploitative”. For example, “Player 1″ is likely to take a “non-exploitative” position while “Player 2″ is likely to take an “exploitative position” in the use of urban green commons based on “information available” and informed by rational thinking (Fig. 3).

**Fig. 4:**
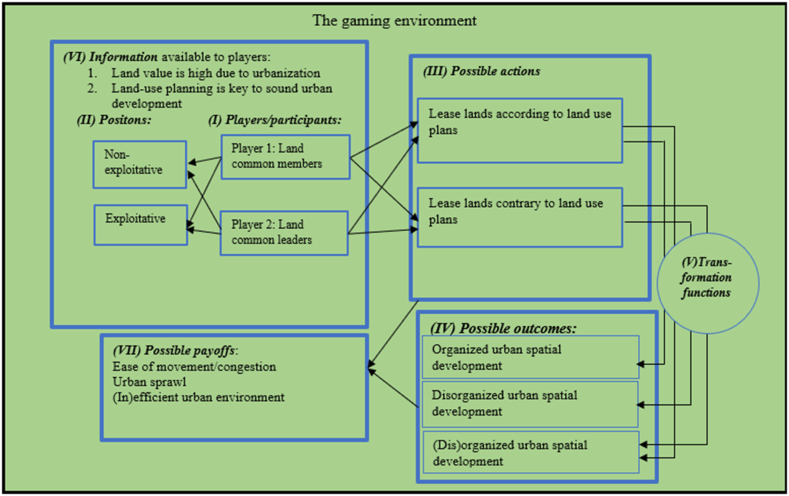
The game of urban land commons through the lens of the IAD framework.

For land commons, we treat the “players” into two groups. They are “customary land ownership members” - land common members regarded as “Player 1" and the “chiefs/clan head/family head” - land common leaders as “Player 2". These players can either take “exploitative” or “non-exploitative” positions depending on the “information available.” For instance, “Player 2" can lease out large acres of land to private real estate developers for a huge amount of profit, without the due engagement of other members as well as non-conformity to urban land use planning schemes (Fig. 4). This is a great possibility as land values are high in urban Ghana, which can entice self-seeking leaders and members to lease land for their own economic gains. Such an action can trigger conflict within the family and lead to poor urban spatial planning outcomes, which are major issues that affect lands in Ghana [75,93].

**Fig. 5:**
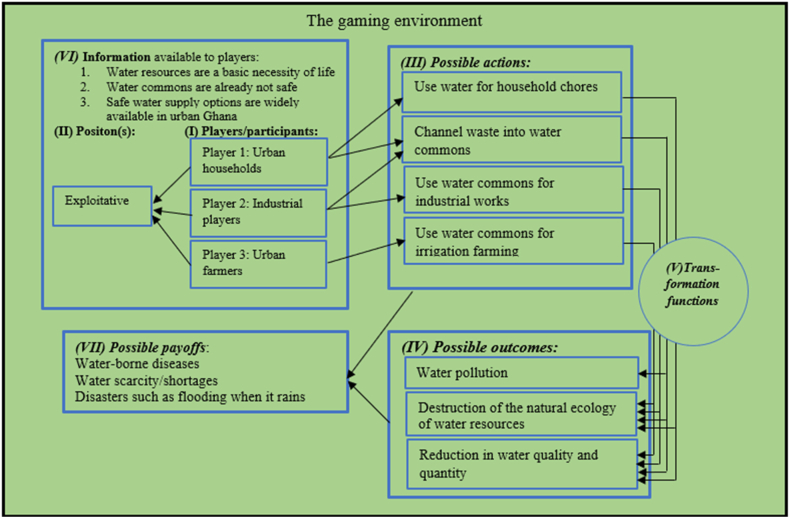
The game of urban water commons through the lens of the IAD framework.

Water resources in urban Ghana are predominantly utilized by three key groups of urban members — “the urban households” who depend on nearby rivers for basic household activities such as washing, bathing, and cleaning; “private industrial players” who utilize such resources for industrial activities; and finally, “urban farmers” who practice, for instance, backyard farming. These actors - urban households, industrial players, and urban farmers - are, in this study, classified as “players” 1, 2, and 3 respectively in the gaming environment for urban water resources management. All the key “players” understand that water is a necessity of life (as “information available”), but their actions are usually detrimental as they hardly care for the ‘well-being’ of the water commons in Ghana [105]. Thus, in the context of water management, the position of the different “players” is largely the same as it is a common notion that neighborhood (common) water resources are not safe for drinking i.e., “information available”, hence, no need to properly care for them. Unlike green commons and land commons, local leaders such as chiefs in the urban context hardly show interest in managing water commons. In the context of piped water supply shortages, urban members have developed local coping mechanisms for treating urban water resources (for instance, rivers) by boiling or through the addition of aluminum sulphate. This practice has somewhat influenced the apathy of urban members (i.e., household members, industrial players, and urban farmers) toward the proper management of urban water commons for the collective good (Fig. 5).

## 7 Conclusion

The conceptual analysis of the three urban commons reveals intense manifestations of tragedies. However, there are instances where the actions of urban members can trigger positive outcomes (for instance, the lease of land in accordance with land use planning provisions for organized urban development). The self-organizing institutions for urban commons management are largely inadequate for commons sustainability. This makes decentralized urban planning agencies relevant to take charge of the management of urban commons. In situations where there are self-organizing institutions (for example, the case of land), collaborative efforts are needed for a robust institutional framework that would protect the urban commons. It is possible to incorporate self-organized institutional arrangements for commons governance into the decentralized governance system [13]. However, urban planning anomalies need to be rectified in Ghana moving forward. Resource availability, recognition of the relevance of urban planning as a technocratic participatory tool for development, and disconnection of planning from political influence are key ingredients that can make urban planning very instrumental for Ghana's development. On this basis, planning can be spearheaded to ensure that the self-centered motives of powerful actors are impeded. The negative payoffs that predominantly surround urban commons can, therefore, be dealt with.

There are, of course, competing interests amongst actors of self-organizing institutions for commons governance. The complexities of these interests can be dealt with through urban planning, which is a problem-solving diagnosis measure to balance the multiplicity of interests for a compromise that puts the commons ‘well-being’ and sustainability first. In the case of green commons, urban planning in Ghana holds the potential to expand such resources in strategic areas for safe urban living. In the context of land commons, the revelation of Cobbinah et al. [75] in meeting the requirements of effective urban land-use planning in Ghana is worth emphasizing. The requirements are a clear definition of the roles of Land Sector Agencies (LSAs) and related institutions, stakeholder engagement and coordination, development planning and control, policies/law enforcement, and the adoption of high densities development. Once these requirements are met, urban planning can be streamlined to sustainably protect land commons in Ghana. It will also be laudable for decentralized water resource planning to be granted attention if Ghana's water resources, especially those in the urban centers can be well protected and managed. This calls for a strategic polycentric governance regime that puts decentralized (urban) planning agencies in a strategic position to complement the efforts of the national-level institutions. Community participation and fostering social interactions are also important factors for the effectiveness of urban planning policies for sustainable management of the urban commons. The manifestation of urban planning failures and the fast waning of urban commons occur in many developing countries, especially the SSA region of Africa, which shares similar urban development problems with Ghana. It, therefore, becomes relevant that the findings of the study are much more applicable to such countries, as the proposals can easily be contextualized for the sustainability of the urban commons in other SSA countries.

**Limitation of the study:** This study is a review of existing urban planning and urban commons literature. As part of our contribution, we were interested in quantifying the degree of depletion of the urban commons considered in the study, as well as the changes in them over time. This could have helped to identify which of the urban commons is following the most unsustainable path and therefore needs intense policy actions/measures. However, due to the descriptive nature of how the urban commons are depleting in the available literature concerning Ghana, it became impossible to do that from a review perspective. Future works on urban planning and urban commons in Ghana should move beyond literature review and synthesis to consider empirical data in specific urban centers to provide quantitative variables and changes in urban commons and context-specific (policy) insight to inform how urban planning can contribute to sustaining the urban commons.

## Funding

This research did not receive any specific grant from funding agencies in the public, commercial, or not-for-profit sectors.

## Author contribution statement

All authors listed have significantly contributed to the development and the writing of this article.

## Data availability statement

Data included in article/supplementary material/referenced in article.

## Additional information

No additional information is available for this paper.

## Declaration of competing interest

The authors declare no conflicts of interest.

## Footnotes

1 In this study, the term urban commons represent three resources - green commons, land commons, and water commons - within the urban space with a collective ownership and management regime and are non-excludable. We are aware that the city itself is a commons as it belongs to many people, and is open to and shared amongst them, but we are interested in some specific resources in the city that are also commons (i.e. green commons, land commons, and water commons).

2 These are individuals with a common interest in the use of urban commons and are likely to take similar decisions concerning the sustainability of the urban commons in a particular situation.
